# Eggshell Particle-Reinforced PVA/GO Hydrogel with Self-Healing Effect

**DOI:** 10.3390/polym18121541

**Published:** 2026-06-21

**Authors:** Banu Esencan Türkaslan, Merve Dogu

**Affiliations:** Department of Chemical Engineering, Faculty of Engineering and Natural Science, University of Süleyman Demirel, Isparta 32260, Turkey; mervdgu@gmail.com

**Keywords:** biomaterials, self-healing, polyvinyl alcohol, graphene oxide, eggshell particle, composite hydrogel

## Abstract

Self-healing biomaterials have attracted significant attention due to their ability to restore structural integrity, extend material lifetime, and reduce maintenance costs without external intervention. In this study, Polyvinyl Alcohol/Graphene Oxide/Eggshell Particle (PVA/GO/ESP) composite hydrogels were synthesized via a freeze–thawing method and characterized using XRD, SEM/EDS, and FTIR analyses. The effect of ESP incorporation on the self-healing and mechanical properties of the hydrogels was systematically investigated. Tensile test results demonstrated that incorporation of 1 wt% ESP improved the tensile strength up to 0.326 MPa while maintaining high strain capacity. Healing efficiency values calculated from recovered tensile strength showed approximately 69%, 47%, and 67% recovery for PVA/GO, PVA/GO/ESP (0.5%), and PVA/GO/ESP (1%) hydrogels, respectively. The developed hydrogels demonstrated rapid self-healing behavior at room temperature without external stimuli. These findings suggest that ESP-reinforced PVA/GO hydrogels may serve as promising candidates for future biomaterial and soft tissue engineering studies. The developed hydrogels demonstrated enhanced tensile strength, rapid self-healing behavior, and promising swelling properties, indicating their potential use in soft tissue engineering and biomaterial applications.

## 1. Introduction

One of the outstanding optimized properties of biological materials such as leather, bone, and wood is self-healing [1]. Self-healing materials with repairing properties are studied extensively in different fields such as biomaterials [2,3], tissue engineering [4,5], conductors [6,7], and surface coatings [8]. It is a known fact that the majority of tissues and organs of living organisms have self-healing properties. Hydrogels, which are soft and wet materials with similar network structure and mechanical properties to soft bio-tissues, are smart materials open to development under studies on damaged soft tissues [9,10,11]. Hydrogels used in biomedical applications need a high level of hardness, toughness, and fracture resistance to maintain their structure. Yet, most hydrogels have low strength and durability values, severely limiting their application in many of the biomedical practices, such as those on scaffolds, cartilages, blood vessels, tendons, and muscles. Therefore, various methods have been developed to improve the self-healing capabilities of hydrogels, focusing on two important aspects of polymer composites and gels (composite gels and hydrogels), including polymer matrix hydrogels [12,13], nanocomposite hydrogels [14,15,16], and dual matrix hydrogels [17]. Most self-healing polymers are amorphous polymers with good elasticity and a reversibly decomposable hydrogen bonding network. These bonds can reversibly combine and dissociate when exposed to heat or light [18,19,20]. Since the discovery of graphene, polymer/graphene nanocomposites have attracted intense interest with their improved mechanical properties. The fact that graphene is insoluble in watery environment or other organic solvents makes it difficult to distribute it homogeneously in the polymer matrix, thus limiting the use of graphene in polymer/graphene nanocomposites [21]. Recent studies have also highlighted multifunctional self-healing hydrogels with improved mechanical robustness and biomedical potential. In addition, carbon nanotubes and graphene derivatives are widely used as reinforcing nanomaterials due to their superior mechanical strength, thermal stability, and ability to improve the electrical and mechanical properties of polymer matrices.

Graphene oxide (GO) is a hydrophilic layered graphene derivative with high mechanical strength, and chemical and thermal stability, containing many reactive oxygen groups [22]. GO-based polymer nanocomposite gels adapt to the structure of the polymer by forming strong covalent bonds between polymer chains and GO layers via in situ polymerization. The mutual diffusion and hydrogen bonding of long polymer chains at the interface of two damaged gel samples, the interaction with each other and with the GO layers, contribute to the regeneration of a unique polymer/GO network [23]. Yanan et al. developed highly biocompatible and accelerated wound healing capabilities of the hybrid hydrogel based on graphene oxide/rose bengal/polyvinyl alcohol with the purpose of obtaining an effective wound dressing for practical application [24]. Hu et al. designed the highly slow-release lubrication properties and self-healing function of composite hydrogels with good mechanical stability based on embedding GO and PEG into PVA hydrogel networks, which provided a facile approach to fabricating a kind of multifunctional composite hydrogel [25].

Studies on eggshell particles (ESPs) are currently being conducted in a variety of fields, including bone healing [26,27], plastic manufacturing [28], coating [29], paper manufacturing [30], and medicine [31]. Reusing waste eggshells has not only a positive impact on the economy and the environment, but it also addresses the unmet clinical needs [32]. Eggshell comprises two shell membranes, which are a foamy cuticle and a calcium carbonate layer [33]. Calcium carbonate (CaCO_3_), magnesium carbonate (MgCO_3_), calcium phosphate (Ca_3_(PO_4_)_2_), hydroxyapatite (Ca_10_(PO_4_)), and organic compounds are the main constituents of the ESP structure [26]. Matrix proteins in the ESP affect the tissue and biomechanical properties of the shell and accelerate the formation of the matrix by controlling the movements of calcium carbonate crystals. The eggshell membrane’s fibers are made up of bioactive substances such as hyaluronic acid, type I, V, and X collagens, glycosaminoglycans, and glycoproteins. This composition of the eggshell membrane is very similar to the extracellular composition of native human cartilage. In light of this, the eggshell membrane might make a good matrix for tissue engineering applications [34].

This study suggests that self-healing, hard, and durable PVA/GO hydrogels doped with ESP, a natural biomaterial with self-healing properties that occurs as waste in large quantities every year, were synthesized by means of the freezing and thawing method. Herein, we utilized the advantages of both the PVA/GO double network (DN) hydrogels in our novel-designed hydrogels and developed a fully physically cross-linked PVA/GO/ESP hydrogel. To the best of our knowledge, this study is the first report for the preparation of a fully physically cross-linked PVA/GO/ESP hydrogel, displaying quick self-healing behavior, efficient strength recovery, and strong mechanical properties. Compared with previous studies, the present hydrogel system utilizes sustainable waste-derived ESP as a bio-filler while simultaneously improving self-healing and mechanical behavior.

## 2. Materials and Methods

Graphite flake (≥75% min), sulfuric acid (H_2_SO_4_, 98%), potassium permanganate (KMnO_4_, 99%), hydrogen peroxide (H_2_O_2_, 30%), hydrochloric acid (HCI, 37%), polyvinyl alcohol (PVA 99%, Mw 146,000–186,000 g/mol) were obtained from Sigma Aldrich company (St. Louis, MO, USA) for the synthesis application.

### 2.1. Graphene Oxide (GO) Synthesis

GO was synthesized via the Modified Improved Hummers Method [22] using graphite in flake structure. Firstly, graphite (2 g) and then KMnO_4_ (6 g) were gradually added into 50 mL H_2_SO_4_ within the ice bath and mixed in a magnetic mixer. Later, 300 mL of deionized water was added to the mixture. In order to stop the oxidation process and remove the impurities in the structure, the mixture was filtered by adding H_2_O_2_ and HCl, respectively. H_2_O_2_ was added to the mixture, and it was mixed until it showed a homogeneous distribution at a temperature not exceeding 40 °C. It was observed that the color of the mixture turned bright brown. Synthesized graphite oxide measuring 1 g was taken into 350 mL of pure water and dispersed for 3 h. After two hours of sonication in order to facilitate the exfoliation of clumped graphite oxide layers on GO layers, the mixture was centrifuged, and GO was produced.

### 2.2. Preparation of PVA/GO (1% w/v) Hydrogel

**Figure 1:**
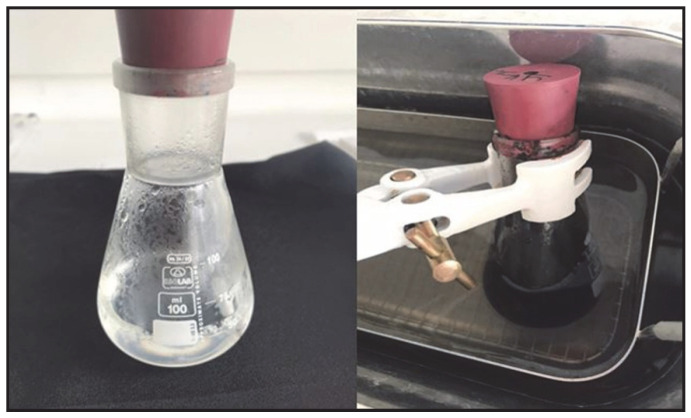
PVA solution and PVA/GO hydrogel.

First, 2.25 g of PVA (99%, Mw 146,000–186,000 g/mol) was dissolved in 15 mL of deionized water and then mixed at 90 °C for 2 h in order to produce a homogeneous solution. Then, 15 mL of GO (1% *w*/*v*) aqueous solution was sonicated in an ultrasonic bath at 60 °C for 30 min and then added to the PVA solution (Figure 1). The solution was sonicated in an ultrasonic bath at 50 °C for 30 min in order to remove the air bubbles formed in the solution, cooled to room temperature.

### 2.3. Preparation of ESP Powder

**Figure 2:**
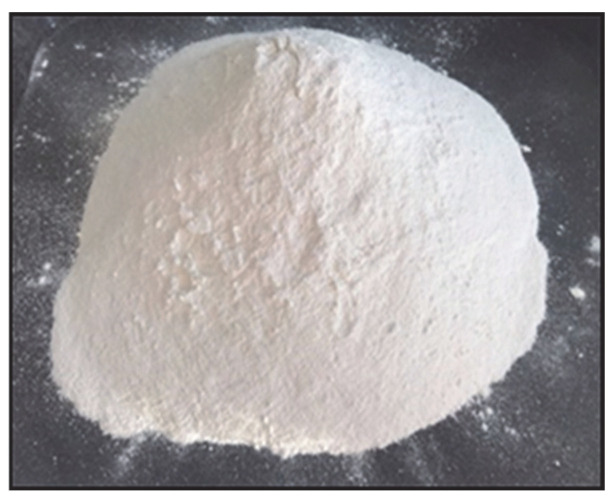
ESP Powder.

ESP was collected from a patisserie for 2 weeks. After the collected eggshells were washed with water and filtered, they were kept in household-type sodium hypochlorite (6%) for 6 h. ESP kept in sodium hypochlorite was washed with water. ESP was dried in the drying oven at 70 °C for 24 h, then ground and sieved to achieve a homogeneous size (Figure 2).

### 2.4. Preparation of PVA/GO/ESP Hydrogels with Different ESP Ratios

Several PVA/GO solutions at the same ratios were prepared to add ESP at different ratios. A total of 2.25 g of PVA was dissolved in 15 mL of deionized water, and it was mixed at 90 °C for 2 h to achieve a homogeneous solution. Then, 15 mL of aqueous GO (1% *w*/*v*) solution was added to the PVA solution and sonicated in an ultrasonic bath at 60 °C for 30 min.

**Figure 3:**
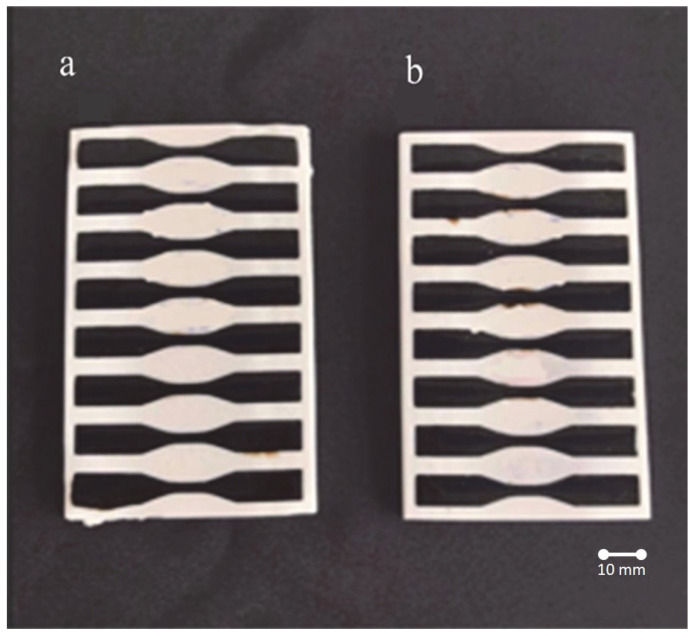
PVA/GO/ESP hydrogels with ESP ratio at (**a**) 0.5% and (**b**) 1%.

After adding 0.5% ESP to one of the prepared PVA/GO hydrogel solutions and 1% ESP to the other, the mixtures were sonicated at 60 °C for 30 min. After being sonicated in an ultrasonic bath at 50 °C for 30 min to remove the air bubbles, the solutions were cooled at room temperature and poured into molds. All samples maintain a geometry characteristic of Standart Test Method for polymer tensile testing ASTM D638 type V [35]. The solutions poured into the molds were subjected to 2 freezing/thawing cycles (freezing temperature: −18 °C, 24 h, thawing temperature: 18 °C, 24 h) (Figure 3).

## 3. Results

The GO characterization was performed with X-ray diffraction (XRD) and the scanning electron microscopy (SEM/EDS) technique. The crystalline phase of GO was examined by X-ray diffraction (XRD, Bruker D8 Advance Twin-Twin; Bruker, Karlsruhe, Germany) at 40 kV, 40 mA, and 1600 watts. PVA/GO/ESP hydrogels were also evaluated by using a scanning electron microscope with a low vacuum at 20.00 kV and 12.7–13.2 mm working distance, respectively (SEM, Quanta Feg 250; FEI, Eindhoven, the Netherlands). The elemental analysis of hydrogels was carried out using an SEM microscope equipped with an energy-dispersive X-ray spectroscopy (EDX, Quanta Feg 250; FEI, Eindhoven, The Netherlands). The distribution and atomic composition of PVA/GO/ESP hydrogels were examined using elemental mappings at an accelerating voltage of 20 kV. Fourier transform infrared (FTIR) spectra (JASCO FT/IR; JASCO Corporation, Tokyo, Japan) were recorded between 400 and 4000 cm^−1^ with a 4 cm^−1^ resolution.

### 3.1. Characterization of GO

**Figure 4:**
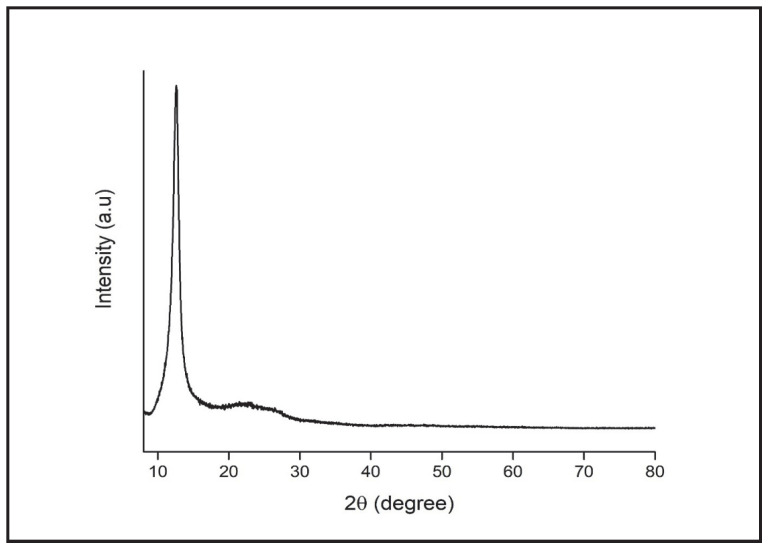
XRD patterns of GO.

A sharp peak of 2θ = 11.8° is observed within the XRD spectrum of the GO structure (Figure 4). The fact that the peak of 2θ = 26.02° existing in the graphite structure is not seen in GO after oxidation, and that a peak of 11.8° was formed instead, is consistent with the results in the literature and shows that the GO structure is obtained properly [23].

The peak assignment (Table 1) provides definite proof for the degree of oxidation, as the layer distance of graphite and GO is very different.

**Figure 5:**
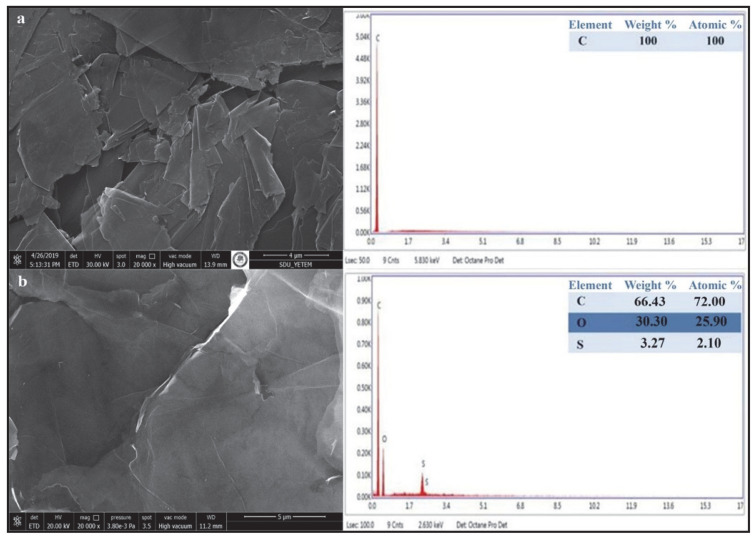
SEM/EDS image of (**a**) graphite and (**b**) GO.

When the results of the SEM analysis are evaluated, it is observed that the GO demonstrates wavy layered structures, positioned on top of each other. Also, in EDX analyses, functional groups were added between the layers as a result of oxidation of the graphite forming the layered GO morphology (Figure 5).

### 3.2. Characterization of ESP

**Figure 6:**
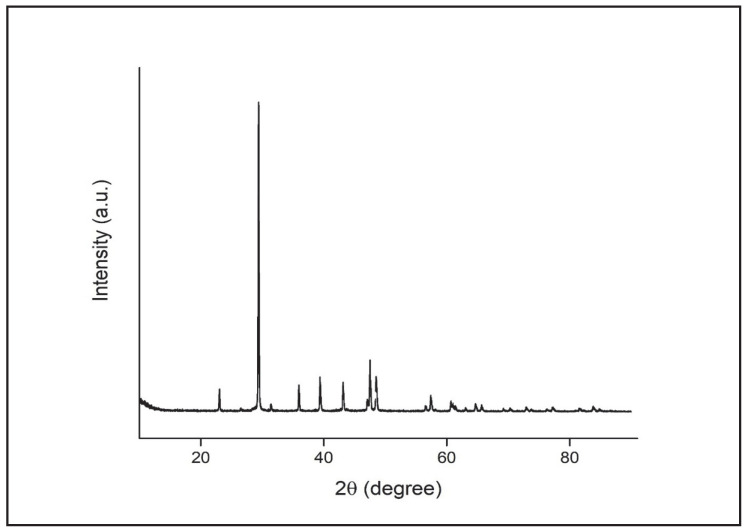
XRD patterns of ESP.

We have observed the peak values of 2θ = 29.4°, 39.4°, 43°, 48.5°, 57.4°, and 65.7°, one of which was sharper, under the scope of observation of the XRD spectrum of the ESP used (Figure 6). These peaks belong to the limestone (CaCO_3_) found in the eggshell structure [36]. In the nanoparticle size measurement of ESP, the particle size was calculated as 60.3 nm.

### 3.3. Characterization of PVA/GO/ESP Hydrogels

**Figure 7:**
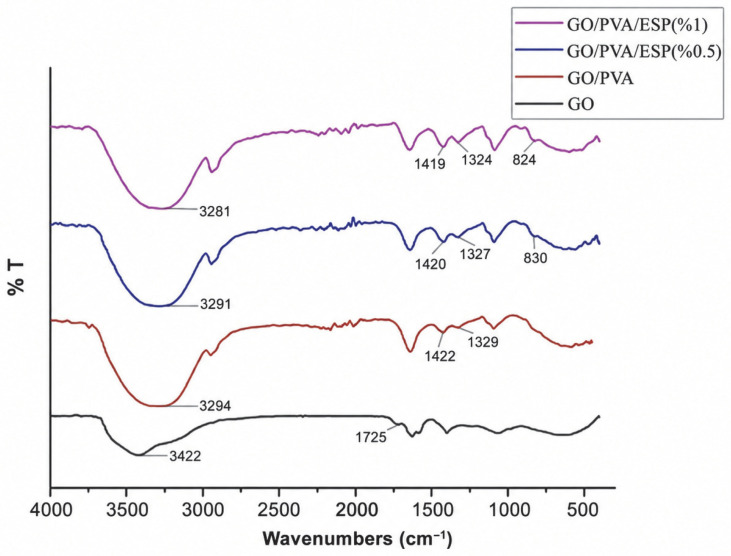
FTIR Spectra of GO, PVA/GO, and PVA/GO/ESP hydrogels.

Figure 7 shows FTIR spectra of hydrogels synthesized at varying ESP ratios. Wide absorption peaks of up to 3300 cm^−1^ belonging to the hydroxyl group were observed in the PVA/GO and PVA/GO/ESP structures. The large number of hydrogen bonds formed between PVA and GO in the double-layer matrix PVA/GO hydrogel structure formed by the addition of GO to the structure shifted the –OH stretching vibrations to lower wavelengths in comparison with the pure PVA hydrogel [24].

The formation of a more severe peak in PVA/GO and PVA/GO/ESP structures with C=O stretching vibration shifted to 1722 cm^−1,^ unlike pure GO, indicates the formation of hydrogen bonds between C=O and -OH [37]. With the addition of GO to the PVA structure, the intensity of the C-H_2_ vibration band observed at 2800–3000 cm^−1^ increased. In-plane bending vibrations of carboxyl groups in PVA/GO and PVA/GO/ESP were seen as two peak levels at around 1320 cm^−1^ and 1420 cm^−1^. The small bands around 827 cm^−1^ and 828 cm^−1^ observed in PVA/GO/ESP composite hydrogels, respectively, belong to the out-of-plane and in-plane deformation vibrations of CaCO_3_ forming the ESP structure, confirming the presence of eggshell in the structure of the PVA/GO hydrogel [24].

**Figure 8:**
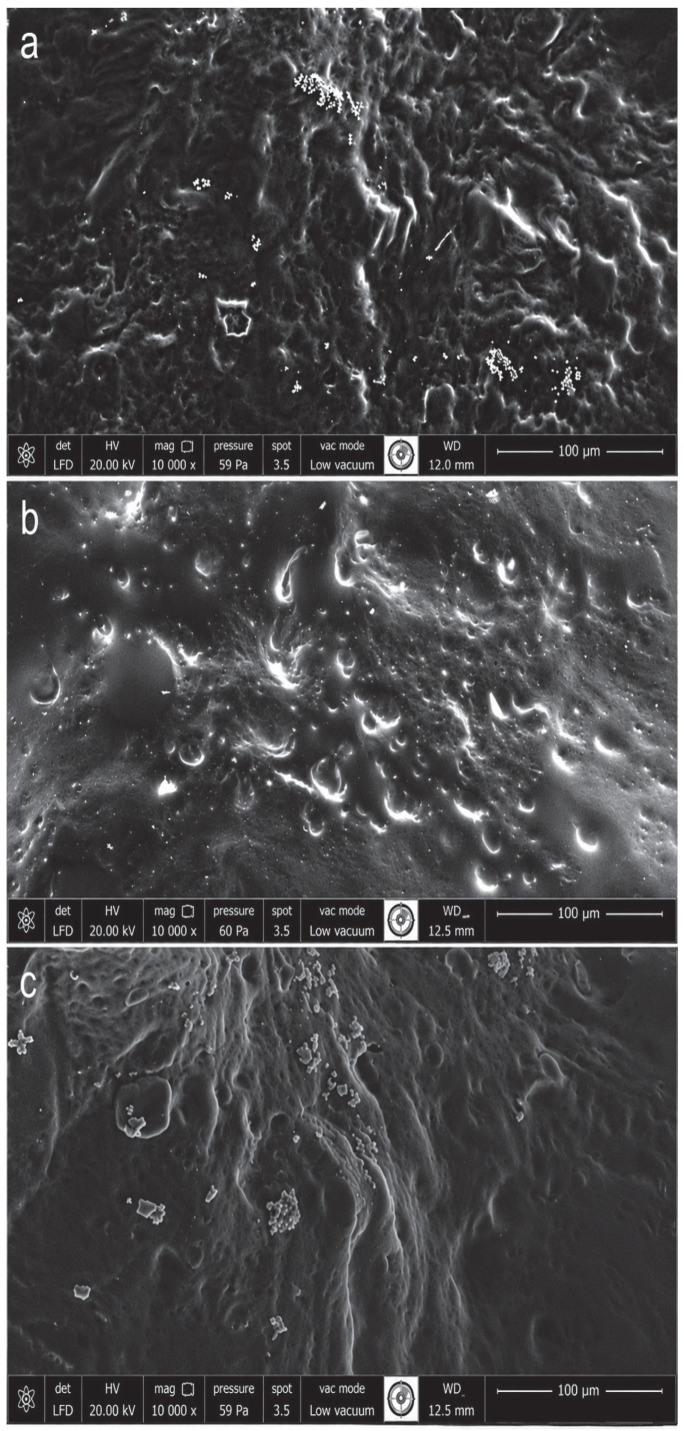
SEM micrographs of (**a**) PVA/GO (**b**) PVA/GO/ESP (0.5%) (**c**) PVA/GO/ESP (1%) Hydrogels.

It was observed that the PVA/GO and PVA/GO/ESP composite hydrogels prepared by the freezing/thawing method have an elastic rubber structure. The SEM images (Figure 8) show that the porous and rough surface of the pure PVA hydrogel turned into a more homogeneous morphology after GO was added (Figure 8a). Additionally, a more non-porous and homogeneous structure was achieved by increasing the amount of CaCO_3_ in the eggshell structure added to the structure of the composite hydrogels at different rates and by intensifying the morphology of the hydrogels (Figure 8b,c). However, these observations are qualitative SEM interpretations only, since quantitative pore size distribution and porosity analyses were not performed. Therefore, the interpretation of morphology was limited to qualitative SEM observations.

SEM examination coupled with an EDX analysis was conducted in order to confirm the presence of calcium on the surface of PVA/GO/ESP hydrogel composites. The compositions of PVA/GO/ESP hydrogel composites are summarized in Table 2. It has been observed that as the amount of ESP increases in the hydrogel structure, the amount of calcium also increases. Variations in elemental composition may originate from local surface heterogeneity, sample preparation conditions, and intrinsic limitations of SEM-EDS analysis.

### 3.4. Self-Healing Efficiency of PVA/GO/ESP Hydrogels

**Figure 9:**
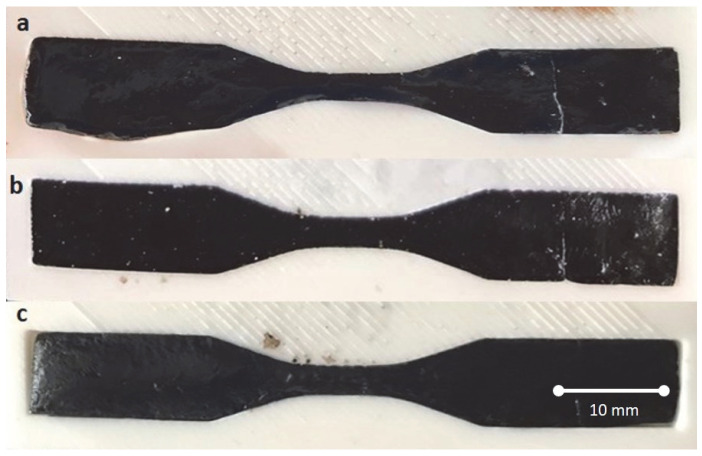
Images of Self-healing (**a**) PVA/GO, (**b**) PVA/GO/ESP (0.5%), and (**c**) PVA/GO/ESP (1%) hydrogels in the molds 10 s after they are joined.

In situ and ex situ self-healing tests were performed to observe the self-healing behavior of PVA/GO/ESP hydrogels. Hydrogels were cut in the middle and combined in the mold without any external stimuli, and the self-healing process was examined at room temperature [38]. Figure 9a–c shows the image of the hydrogels assembled in the mold after 10 s. In order to observe the joining efficiency of the composite hydrogels better, hydrogels were extended from both ends with the help of tweezers, and no detachment was observed at the specimens without any external force at the self-healed area. (Figure 9a–c) [39].

The self-healing performance of the hydrogels was visually and mechanically evaluated. Representative photographs of the healing process demonstrated that the separated hydrogel pieces rapidly rejoined within a short period at room temperature without requiring any external stimulus. After reconnection, the healed hydrogels maintained structural integrity during manual stretching tests, confirming successful interfacial network reconstruction through reversible hydrogen bonding interactions between PVA, GO, and ESP components.

### 3.5. Mechanical Test Results of PVA/GO/ESP Hydrogels

In order to further evaluate the self-healing property quantitatively, tensile tests were performed on the synthesized original hydrogels and the self-healing hydrogel samples, and stress–strain curves were compared. The tensile test was performed using the Shimadzu AGS-X tensile device with a load of 10 kN and a tensile speed of 10 mm/min. All experiments were conducted at least in triplicate to ensure reproducibility. However, detailed statistical analysis and confidence interval calculations were not included within the scope of the present study.

**Figure 10:**
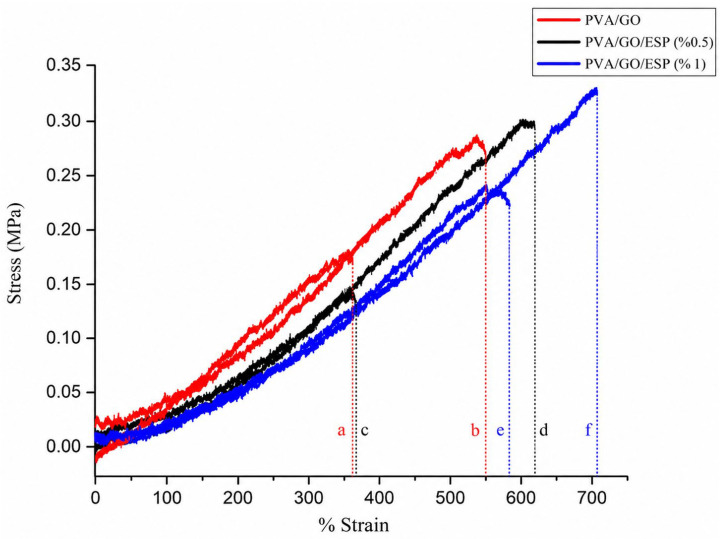
Tensile graphs of after self-healing process (a) PVA/GO (c) PVA/GO/ESP (0.5%) (e) PVA/GO/ESP (1%) and in their original phase (b) PVA/GO (d) PVA/GO/ESP (0.5%) (f) PVA/GO/ESP (1%).

Figure 10 shows the compressive stress–strain curve of the PVA/GO hydrogel solutions at the same ratios, which were prepared with different ESP dosages (0.5%, 1%). The maximum stress of the PVA/GO composite hydrogel was 0.255 MPa, and the strain was read as 551.151%. The increased stress value of the PVA/GO hydrogel is due to the orientation of the GO layers in the matrix structure. During the strain process, the GO layers align along the strain direction due to the hydrogen bonds present between the hydroxyl and carboxyl groups, and so when the hydrogel is strained, the GO layers are also strained, leading to an increase in the strain value [40].

After the addition of 0.5% ESP, the strain value increased to 606.281% and the stress to 0.299 MPa. Given these values, the ESP reinforcement material, which was increased by 1% in weight, increased the strain value from 551% to 706.80% compared to PVA/GO hydrogel, and the amount of stress corresponding to this strain value reached up to 0.326 MPa. The increase in the amount of ESP added to the hydrogel had a positive effect on the tensile strengths. The lower tensile strength observed after healing for the PVA/GO/ESP (0.5%) hydrogel may result from insufficient ESP content for effective stress transfer and interfacial network recovery during the self-healing process. This enhancement may be attributed to improved interfacial interactions, hydrogen bonding, and efficient stress transfer between ESPs, GO nanosheets, and the PVA polymeric network.

Results of tensile tests performed following the self-healing processes showed the maximum tensile strength of the PVA/GO composite hydrogel as 0.176 MPa and the maximum strain value as 351.469%. Healing efficiency (η) was quantitatively evaluated using the ratio of the tensile strength of healed hydrogels to the original tensile strength according to the Equation:

η (%) = (σ_healed_/σ_original_) × 100.

Based on tensile test results, healing efficiency values were calculated as approximately 65% for PVA/GO hydrogels, 60% for PVA/GO/ESP (0.5%) hydrogels, and 82% for PVA/GO/ESP (1%) hydrogels. These results confirm that ESP incorporation contributes positively to the recovery of mechanical performance after damage. The obtained healing efficiencies demonstrate that the incorporation of 1 wt% ESP maintains healing capability while simultaneously improving the mechanical strength of the hydrogel system.

After the addition of ESP reinforcement material at the ratios of 0.5% and 1%, tensile strengths were observed as 0.14 MPa and 0.22 MPa, respectively. The 0.5% values were lower; however, 1% values were higher than the PVA/GO hydrogel. However, the strain values were found to be 361,869% and 489.26%, where both values are recorded better than the PVA/GO hydrogel. After the self-healing property, the highest tensile strength and tensile value were observed in PVA/GO/ESP hydrogel with 1% ESP. It has been observed that ESP added to the PVA/GO hydrogel structure improves the self-healing efficiency of the hydrogel as well as its mechanical properties.

### 3.6. Water Content of Hydrogel

The samples were dried in a drying oven at 50 °C for 30 min to measure the water content of the prepared hydrogels. Equilibrium water content W_0_ is the weight of the sample before drying, W is the weight of the sample after drying, as follows. Although this method enabled comparative evaluation between samples, complete water removal may not have been achieved due to the highly hydrophilic nature of PVA-based hydrogels. Therefore, future studies will employ lyophilization (freeze-drying) techniques to obtain more accurate equilibrium water content values.

Equilibrium water content ratio (%) = (W_0_ − W)/W_0_ × (100%)

**Figure 11:**
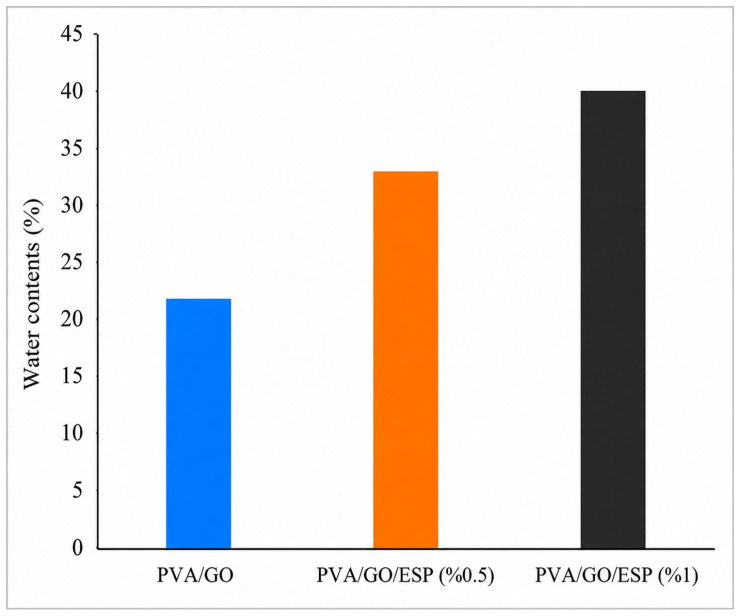
Water contents of PVA/GO and PVA/GO/ESP hydrogels.

Observations on the water contents of the hydrogels containing PVA/GO and different ratios (0.5% and 1%) of ESP in Figure 11 show that the water content of the hydrogels with ESP was higher than that of the PVA/GO hydrogel. It is seen that this ratio, which changes with the increasing amount of ESP in the hydrogel structure, is due to the water interaction behavior of calcium-containing composite structures in the composition of the ESP.

### 3.7. Swelling Ratio of Hydrogel

Hydrogel samples were dried in an oven at 50 °C until they reached constant weight to determine the effect of ESP on swelling behavior. The samples were then immersed in sufficient distilled water until completely submerged. When equilibrium was reached, the surfaces were wiped with filter paper before the samples were removed from the water and weighed. The swelling calculation was performed as follows (W_0_ shows the weight before soaking, and W shows the weight after soaking in water).

Swelling rate (%) = (W − W_o_)/W_0_ × (100%)

**Figure 12:**
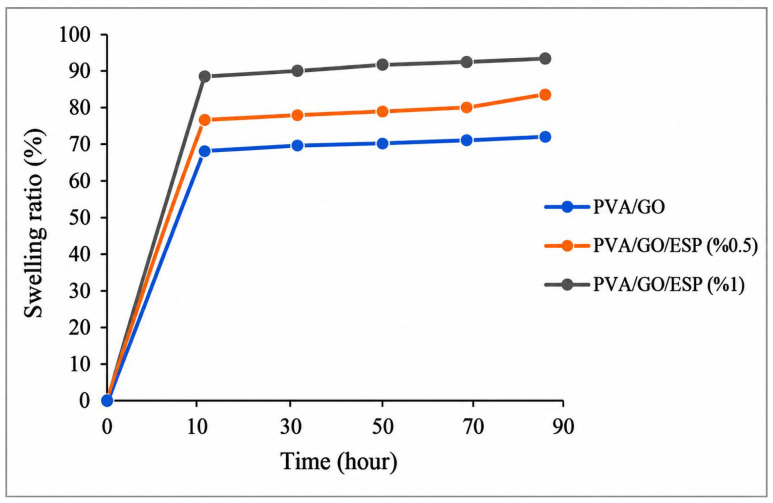
Swelling ratio of PVA/GO and PVA/GO/ESP hydrogels.

Figure 12 shows that hydrogels had similar swelling rates. It has been determined that the functional groups in the structure of GO (such as -COOH, and -OH) cross-link with PVA macromolecules and form hydrogen bonds, and ESP added in the hydrogel increases the swelling rate in the PVA/GO hydrogel structure [39].

## 4. Discussion

PVA/GO/ESP composite hydrogels were prepared by a freeze/thaw method with various ESP ratios. It has been observed that PVA/GO/ESP composite hydrogels were able to self-repair with the cut surfaces in contact at room temperature for a short time without the need for external stimuli. Moreover, PVA/GO/ESP hydrogel demonstrated not only rapid and efficient self-healing behavior but also strong mechanical properties. The healed PVA/GO/ESP hydrogels exhibit the highest tensile strengths (up to 0.326 MPa) when 1 wt% of ESP was incorporated into the PVA/GO. The large surface area of GO within the matrix and strong interactions between the PVA and the GO sheets, as confirmed by SEM and FTIR, are the key factors for the rapid self-healing ability and strong mechanical properties of the developed gel.

## 5. Conclusions

The developed PVA/GO/ESP hydrogels exhibited tensile strengths up to 0.326 MPa and healing efficiencies approaching 67–69%. These characteristics indicate potential applications in flexible biomaterials, soft tissue engineering, wound dressing, and self-healing scaffold systems.

We reported a chemical synthesis process and the self-healing ability of PVA/GO/ESP hydrogels. We observed that adjusting different ESP concentrations is an important parameter for self-healing ability and mechanical properties of the hydrogel. This is attributed to the fact that calcium carbonate, one of the ESP components in the structure, increases the regeneration of hydrogels with its superior biophysical properties.

The current study mainly focuses on the investigation of self-healing hydrogel through the synthesis of PVA/GO/ESP. Even though the production of ESP-reinforced PVA/GO hydrogels was confirmed by FTIR, SEM, and mechanical test results, further study in the aspect of potential reaction mechanisms, the purity of the product, and optimum parameters needs to be investigated for many biomedical applications such as scaffolds, cartilages, tendons, and muscles. In addition, no cytotoxicity or in vitro biocompatibility analyses were performed within the scope of the present study. Therefore, although the developed hydrogels demonstrate potential for biomedical applications, comprehensive biological evaluations, including MTT, Live/Dead, and cell viability assays, are required in future studies to validate their suitability for tissue engineering applications.

## Figures and Tables

**Table 1:** Peak assignment of graphite and GO.

Sample	2 (°)	d (nm)	Number of Layers
Graphite	26.02	0.34	47
GO	11.8	0.82	14

**Table 2:** Elemental composition.

	Element	wt%
PVA/GO	C	48.50
	O	51.50
PVA/GO/ESP (0.5%)	C	62.35
	O	37
	Ca	0.65
PVA/GO/ESP (1%)	C	68.71
	O	29.28
	Ca	2.01

## Data Availability

All data analyzed during this study are included in this published article.
